# Cell type heterogeneity in gene co-expression networks: implications for toxicological research

**DOI:** 10.1093/bib/bbaf421

**Published:** 2025-08-26

**Authors:** Imke B Bruns, Yingxue Li, James L Stevens, Bob van de Water, Giulia Callegaro

**Affiliations:** Division of Cell Systems and Drug Safety, Leiden Academic Centre for Drug Research, Leiden University, Einsteinweg 55, 2333 CC Leiden, The Netherlands; Division of Cell Systems and Drug Safety, Leiden Academic Centre for Drug Research, Leiden University, Einsteinweg 55, 2333 CC Leiden, The Netherlands; Division of Cell Systems and Drug Safety, Leiden Academic Centre for Drug Research, Leiden University, Einsteinweg 55, 2333 CC Leiden, The Netherlands; Division of Cell Systems and Drug Safety, Leiden Academic Centre for Drug Research, Leiden University, Einsteinweg 55, 2333 CC Leiden, The Netherlands; Division of Cell Systems and Drug Safety, Leiden Academic Centre for Drug Research, Leiden University, Einsteinweg 55, 2333 CC Leiden, The Netherlands

**Keywords:** gene co-expression analysis, (sc-)RNA-seq, cell type heterogeneity, toxicogenomics

## Abstract

A fundamental goal of biological research is to determine the interactions and functional relationships between genes and their coded proteins that drive biological responses. Understanding the response of the global transcriptome in the context of pathogenesis and drug-related adversities can reveal gene–response relationships that contribute to biogical insights and more accurate and reliable mechanism-based safety assessments. Although transcriptomic data provide a framework to systematically determine gene activity, their high dimensionality and complexity can make interpretation and analysis challenging. Gene co-expression analysis addresses these difficulties in analyzing transcriptomics data by first constructing networks of genes that are co-expressed across treatments, reducing complexity, and then inferring biological relevance and gene–pathology associations for each network. Variation in gene expression in bulk tissue helps define co-expression relationships, but the cell type heterogeneity, inherent to bulk tissue, can also complicate biological interpretations. Consequently, interpretation of some tissue gene co-expression patterns may be subject to the confounding influence of variations in cellular composition obscuring intra-cell-type-specific co-expression network responses. In this review, we highlight methods designed to capture cell type–specific co-expression patterns and discuss their potential utility for understanding mechanisms of toxicity and pathogenesis.

## Graphical Abstract

**Graphical Abstract:**
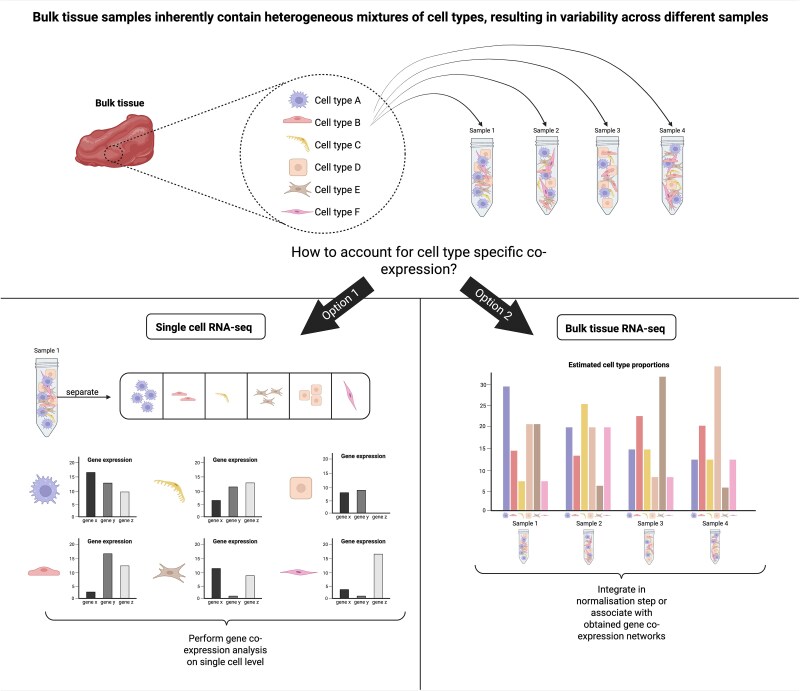

## Introduction

An essential objective in biological research is to derive comprehensive mechanistic understanding of disease pathogenesis, including unveiling mechanisms of adverse drug reactions and toxicity [1]. Tissue pathologies are characterized by inherent complexity and multifactorial nature as genes, proteins, and many other individual players act in networks at the intra- and intercellular levels [2]. Systems biology approaches to disease seek to elucidate pathophysiological trajectories by determining the interactions and functional relationships between e.g. genes and their coded products, within the context of a complex tissue response [3]. Omics technologies are an important data layer for systems biology approaches: in particular, transcriptomics data provide a reasonable estimation of global molecular processes [4] and are widely available thanks to the development of high-throughput technologies [5, 6].

Gene co-expression analysis facilitates systems-level analysis using the variation in expression across groups of samples and treatments to construct networks of genes that are co-expressed [7]. This method assumes genes that are co-expressed under different conditions to operate through similar transcriptional programs and be, therefore, likely functionally related and useful to identify novel gene functions and pathways [8]. Co-expression analysis also enhances understanding of regulatory networks driving both physiological and pathological processes, including transcription factor binding, ligand–receptor pair interactions, and causal regulation of downstream targets [9–12]. In toxicology, gene co-expression is particularly valuable since exposure to substances can trigger numerous interconnected stress and adaptive responses in cells and tissues—many of which depend on transcriptional control [13]. Several studies have utilized co-expression analysis to reveal early-stage biomarkers for chemical-induced toxicity [14], characterize the associations between gene and phenotypes [15, 16], and study the translation of network information from nonclinical to clinical models [17]. All these studies demonstrate the value of co-expression analysis in dealing with large-scale transcriptomics and toxicogenomics data.

A constructed gene co-expression network depends on the variation in the underlying gene expression to identify groups of genes that tend to change together. For network generation, increasing both the number of samples and the variety of treatment conditions (e.g. varying time points and doses) broadens the range of biological processes represented, thereby improving the capture of transient associations. Moreover, gene co-regulation patterns may change due to interactions that might only exist under a specific condition not captured with limited data. Indeed, a previous study showed that co-expression networks clearly benefit from increasing sample size and variation in expression, while performance of small sized datasets (*n* < 100) was variable due to the limited range provided by the small sample set [18].

Importantly, many gene co-expression studies rely on data derived from bulk tissue samples composed of many cell types, each with distinct functions and associated transcriptional profiles. As a result, these measurements represent an averaged expression level across all the different cell types present. This may obscure genuine co-expression relationships, introduce noise, and ultimately lead to misleading conclusions [19, 20]. Moreover, variation in cell type abundance—a key source of expression variation in bulk samples [21, 22]—further complicates network interpretations and can significantly influence the resulting co-expression patterns and hamper the discovery of important biological responses.

So far, no comprehensive review has examined how cell population heterogeneity influences gene co-expression networks and how this can be addressed when investigating the pathogenesis of drug-induced tissue injury. More specifically, this issue has been largely overlooked in co-expression analyses for drug and chemical toxicogenomics studies [1]. Yet, pinpointing the specific cell types susceptible to toxicity would help identify primary target cells and guide more informed decisions about exposure limits, safety regulations, and risk management strategies. Herein, we review different methods to identify cell type–specific co-expression patterns and applications to understanding mechanisms of toxicity and pathogenesis.

## Impact of cell type heterogeneity on co-expression patterns

Cell type abundance (the relative quantity of a given cell type) and heterogeneity (the range of distinct cell types)—collectively termed composition—may influence how co-expression networks form. For example, a significant change in the abundance of a minor cell type within a heterogenous population may lead to shifts in network structure. While several studies suggest that variation in tissue composition can indeed impact gene co-expression analysis [23–25], others report that core co-expression signals remain largely consistent across different cell types as shown by Harris [26], Crow [27], and colleagues.

One crucial factor to consider is the cell type–dependent gene expression levels. Harris *et al*. only selected genes with high average expression levels across different cell types from single-cell expression datasets [26]. However, restricting the analysis to genes with consistently high expression levels overlooks the possibility that certain genes may be specific to particular cell types, thus preventing the identification of cell type–specific co-expression patterns. As a result, their conclusion that core co-regulatory networks are consistent across different cell types primarily could reflect genes that are universally expressed, such as housekeeping genes.

In agreement with this, Farahbod and Pavlidis also found that co-expressed clusters of housekeeping genes from bulk tissue were reproducible in the single-cell population [23]. However, when considering the whole transcriptome, comparison of co-expression clusters formed in single-cell and bulk tissue indicated that intra-cell-type co-expression patterns observed in single-cell data can be masked in bulk tissue by the effect of cellular composition. For example, in a recent study, 33% of the co-expressed genes were observed in both bulk tissue RNA-seq and scRNA-seq co-expression networks [28]. While most enrichments detected in the heterogeneous bulk tissue network also appeared in the single-cell co-expression networks, the additional enrichments of cell-type-specific, low-expression mRNAs in the single-cell data indicate a superior capacity to capture more specific co-expression relationships.

Another source of mismatch between bulk and single-cell networks is spurious co-expression caused by genes that are highly expressed in one cell type, but are not co-regulated, and vary with the proportion of this specific cell type in the bulk sample [29]. For example, an influx of inflammatory cells associated with disease processes would increase the presence of genes annotated for processes associated with inflammation [30], but those genes are not necessarily co-regulated at the level of specific lymphocytes. As an illustration in a toxicity study, Sutherland *et al.* identified a co-regulated network annotated as an immune system process [15]. This network encompasses diverse immune-related genes that are highly expressed in lymphocytes, creating the appearance of co-expression. This apparent co-expression is likely influenced by fluctuations in the levels of inflammatory cell influxes: when there is an increase in inflammatory cells, the expression of these genes rises together. In essence, the observed co-expression in the broader network may be a result of the overall changes in cell populations, and investigating co-expression patterns at cellular level could unveil distinct pathways within specific inflammatory cells. For example, a mouse-lung toxicogenomics study combining single-cell RNA-seq with SCENIC found that a recruited monocyte-derived macrophage subset emerges after lipopolysaccharide induced acute lung inflammation, characterized by strong activity of the Cebpa, Runx3, and Irf5 regulons, which are virtually inactive in the resident interstitial macrophages [31]. For example, a mouse-lung toxicogenomics study combinding single-cell RNA-seq with SCENIC - an algorithm that reconstructs transcription factor-target networks - found that a recruited monocyte-derived macrophage subset [32]. In bulk lung RNA-seq, these regulon-controlled genes may appear tightly co-expressed, but single-cell resolution reveals that this pattern reflects the appearance of a transcriptionally distinct macrophage population. The example further illustrates how network-level correlations can be arise from changes in cell composition rather than true co-regulation, highlighting the importance of modeling cell-type heterogeneity in toxicogenomic studies.

**Figure 1:**
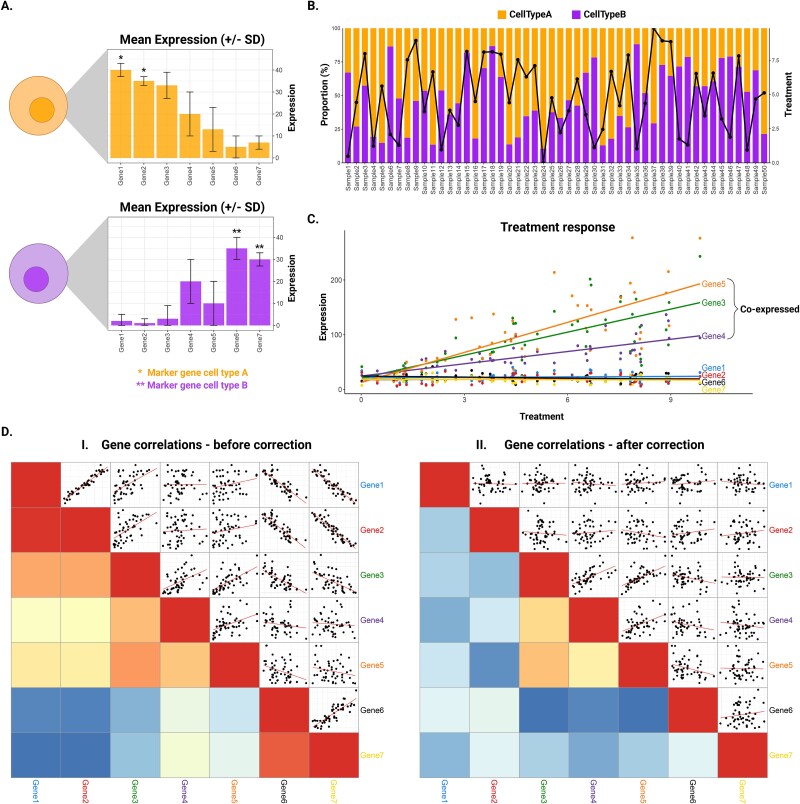
Cellular composition and gene expression level effects on gene co-expression analysis. Bulk sequencing samples often vary in their cellular composition, which can confound co-expression patterns. In this simplified example, 50 samples were simulated to vary in both cell type composition (A versus B, shown in stacked bars in panel B) and an added treatment factor ranging from 0 to 10 (black line in panel B). Genes 3, 4, and 5 respond to treatment and are therefore expected to co-express (panel C). However, large shifts in cell-type proportions cause spurious correlations among cell-type marker genes (Gene 1 + 2 and Gene 6 + 7 in panel D_I_). After correcting the expression data for the varying cell-type proportions, these artifactual correlations disappear, leaving only the true-positive co-expression patterns driven by the treatment factor (Gene3 + Gene4 + Gene5 in panel D_II_).

To further demonstrate this concept and its implications for toxicity studies, we generated a synthetic dataset of 50 samples with varying proportions of two cell types (cell type A and B) and seven genes (Fig. 1, Supplemental Fig. 1). A treatment response was specifically introduced for Gene3, Gene4, and Gene5 (Fig. 1C) to highlight how true co-expression patterns can be obscured by variations in cell type proportions. Notably, we observed strong correlations between Gene1 and Gene2, as well as between Gene6 and Gene7, which are marker genes for Cell Types A and B, respectively. In contrast, the expected co-expression patterns between Genes 3, 4, and 5 due to a similar treatment response were much weaker, highlighting how variations in cell-type composition can obscure true expression relationships (Fig. 1D i).

Although several studies have illustrated the value of co-expression network analysis in elucidating meaningful mechanistic interpretations during tissue injury [15, 16, 33], the impact of cell composition on gene co-expression in heterogeneous tissues has not been assessed in the context of toxicological studies. A study by Wijaya *et al.* concluded that the activation of co-regulated gene networks was divergent in different parts of the kidney, aligning with Lake *et al*.’s kidney atlas, which revealed varying expression profiles in diverse cell types under different conditions [34]. Moreover, they found that whole kidney responses to nephrotoxicants, which commonly target the proximal tubule epithelium [35], were less sensitive than in the proximal tubules, suggesting that bulk sequencing might miss responses in specific parts of the kidney [36]. Taken together, these findings indicate the need to consider cell type–specific expression patterns when constructing systems biology models for toxicological applications. To this end, we herein discuss different approaches and methods to tackle the problem of cell heterogeneity in analyzing transcriptomic data.

## Methodologies to identify cell type–specific co-expression networks

**Figure 2:**
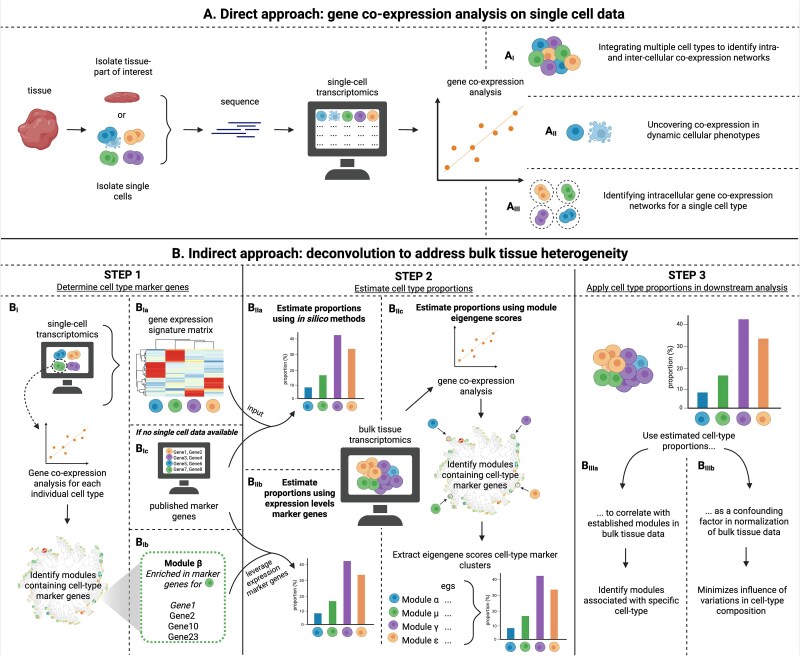
Current applications to identify cell type–specific co-expression networks. There are currently five approaches to identify cell type–specific networks, which can be roughly divided into two overarching methodologies (panel A and B). The first three approaches involve separating cell types from bulk tissue, either through spatially informed methods such as laser capture microdissection followed by RNA-sequencing (LCM-seq), or through cell sorting, followed by direct application of a gene co-expression analysis to data on single-cell level (panel A). This can be performed on all cell types simultaneously, by integrating multiple cell types to identify intra- and intercellular co-expression networks (A_I_) on a specific cell type in different states to uncover co-expression in dynamic cellular phenotypes (e.g. healthy/diseased cell) (A_II_), or a single-cell type to identify intracellular gene co-expression patterns (A_III_). A second general methodology aims to account for the cell-type proportions in bulk tissue samples (panel B). This multistep approach leverages cell-type proportions derived from bulk RNA-seq data. STEP 1: Cell type–specific markers are essential to estimate proportions. These markers can be identified form single-cell RNA-seq data (B_Ia/b_) or obtained from publicly available resources (B_Ic_). STEP 2: Marker genes (or a full gene expression signature matrix from single-cell transcriptomics) can then be applied to estimate cell-type proportions using *in silico* methods (B_IIa_) or by leveraging the expression levels of top marker genes for each cell type (B_IIb_). Alternatively, gene co-expression analysis of bulk RNA-seq data can identify modules enriched with cell-type markers. The first principal component of such a module (the module eigengene score) reflects the relative abundance of the associated cell type (B_IIIc_). STEP 3: The derived cell-type proportions can inform downstream analyses. One approach is to correlate co-expression modules with cell-type proportions to identify modules strongly associated with specific cell types (B_IIIa_). Another approach is to use these proportions as confounding factors during normalization to minimize the variability from cell-type composition in bulk tissue samples (B_IIIb_). Figure generated using BioRender.

Currently, there are five types of approaches to untangle the intracellular co-expression patterns that can be divided into two overarching strategies—direct single-cell gene co-expression analysis and indirect deconvolution from bulk tissue expression patterns (Fig. 2, panels A and B). The direct approach involves separating cell types from bulk tissue and then applying gene co-expression analysis at the single-cell level (Fig. 2, panel A). This can be done in several ways: (1) analyzing all cell samples simultaneously (Fig. 2A_I_); (2) focusing on one specific cell type that can take on two different states (e.g. healthy/diseased cell) (Fig. 2A_II_>); or (3) conducting individual co-expression analyses for each cell type separately (Fig. 2A_III_). In the second general strategy, indirect deconvolution from bulk tissue data, marker genes are identified (Fig. 2, panel B_I_) and used to estimate the cell-type proportions in a heterogeneous bulk tissue sample (Fig. 2, panel B_II_). Subsequently, these proportions can be applied in a downstream step to control for cell-specific contributions to the transcriptomic responses (Fig. 2, panel B_III_). One approach to downstream deconvolution is to apply the estimated cell-type proportions as a confounding factor in the normalization step (Fig. 2B_IIIa_). A second approach is to identify gene co-expression networks derived from the bulk tissue transcriptomic data that are strongly correlated with the presence of specific cell types based on the marker genes (Fig. 2B_IIIb_). These approaches, together with studies applying them and their outcomes, are summarized in Table 1, and their strengths and weaknesses are addressed below.

**Table 1:** Overview of the methods and outcome of the studies evaluated in this review

Method		References	Algorithm	Code availability	Outcome
Co-expression analysis on single cell data (all cell types together)		He *et al.*, 2024	SCENIC	Upon request	Cell type–specific modules
		Cao *et al.*, 2023	WGCNA	N/A	
		Luo *et al.*, 2015	WGCNA	N/A	Mixed modules
		Panwar *et al.*, 2021	mWGCNA	https://github.com/ay-lab/SLE-mWGCNA	
Co-expression analysis on single-cell data (same cell type, different states)	scRNA-seq or snRNA-seq	Salehi *et al.*, 2021	WGCNA	https://github.com/nasalehi/scRNAseq_spermatogenesis	Cell state–specific modules
		Xue *et al.*, 2013	WGCNA	N/A	
		See *et al.*, 2017	WGCNA	N/A	
		Al-Dalahmah *et al.*, 2020	MEGENA	N/A	
		Wu *et al.*, 2018	WGCNA	N/A	
	LCM	Y. Wang *et al.*, 2021	WGCNA	N/A	Mixed modules (associated with both healthy and diseased cells)
		Sæterstad *et al.*, 2022	WGCNA	N/A	
	Spatial transcriptomics	Yamada *et al.*, 2022	WGCNA	https://github.com/firstheart123/spatiotemporal_heart	Cell state–specific modules
Co-expression analysis on single cell data (separately on each cell type)		Ye *et al.*, 2020	WGCNA	N/A	Cell type–specific modules
Associate co-expression networks generated with bulk tissue data with cell-type proportions		Lee *et al.*, 2022	WGCNA	N/A	Mixed modules (although associated with the proportions of a specific cell type)
		Pan *et al.*, 2023	WGCNA	N/A	
		Tan *et al.*, 2021	WGCNA	N/A	
		Zhong *et al.*, 2024	WGCNA	Upon request	
		Lim *et al.*, 2025	WGCNA	N/A	
Normalize bulk tissue data on cell-type proportions before applying co-expression analysis		Lin *et al.*, 2021	WGCNA	Upon request	Mixed modules (although normalized for influence of cell type heterogeneity)

Including which of the five identified approaches was used to identify cell type–specific co-expression (analyzing single-cell data from all cell types together, single-cell data within one cell type under different states, single-cell data for each cell type separately, associating bulk tissue co-expression networks with cell type proportions, or normalizing bulk tissue data for cell-type proportions prior to analysis). The “outcome” column indicates whether the resulting modules were cell-type specific or mixed. The “algorithm” column lists the co-expression methods used: WGCNA (scale-free weighted gene networks), mWGCNA (a multilayer extension of WGCNA in which each layer is labeled by cell type), MEGENA (multiscale clustering of planar filtered networks), or SCENIC (inference of transcription-factor regulons). Finally, if the authors provided reusable code, a link is given in the “code availability” column.

### Direct approach: gene co-expression analysis on single-cell data

To focus the analysis on a specific cell type, one can apply a gene co-expression analysis directly to single-cell RNA data (scRNA-data) or spatial transcriptomics data obtained from tissue sections using e.g. laser capture microdissection (LCM) [28]. Separating cells into single-cell populations is a first step in the direct approach. If single-cell data are available, gene co-expression analysis can be applied to multiple different cell types simultaneously (Fig. 2A_I_), to one specific cell type with different phenotypes (Fig. 2A_II_), or to each individual cell type separately (Fig. 2A_III_). The choice of analysis approach depends on the hypothesis being tested. If the goal is to identify patterns that are shared among multiple cell types or arise from intercellular interactions between them, then applying a gene co-expression analysis to all relevant cell types at once is recommended. In contrast, when studying co-expression patterns tied to a specific intracellular process or shifts in cellular phenotype, focusing on a single cell type, or the same cell type in two distinct states (e.g. quiescent or activated stellate cells in the liver), may be more appropriate. Lastly, when investigating co-expression relationships unique to each cell type, the analysis better be performed on each cell type separately. The various approaches to conduct a gene co-expression analysis using single-cell or tissue micro-sampling techniques are summarized in Fig. 2A and will be elaborated upon in the following sections.

#### Integrating multiple cell types to identify intra-and intercellular co-expression networks

The first direct approach involves integrating single-cell data from multiple cell types into a unified dataset to identify both intra- and intercellular co-expression networks (Fig. 2A_I_). This approach is ideal to analyze minor cell populations that would generally be overshadowed in bulk tissue RNA-seq data and allows one to directly test two fundamental hypotheses: (1) are there cell type–specific gene co-expression networks (also called modules)?; or, (2) can mixed modules be found, indicating that co-expression patterns are shared across cell types, tissue sections, and states?

He *et al.* applied SCENIC—an algorithm that reconstructs transcription factor-target networks and quantifies their activity specifically at the single-cell level [32]—to scRNA-seq data obtained from multiple cell types and identified tissue-specific co-expression modules [37]. While long non-coding RNAs (lncRNAs) are often tissue or condition specific and can be obscured in bulk tissue expression data [38], applying gene co-expression analysis directly to single-cell data enabled the functional annotation of these lncRNAs by linking them to associated coding genes [37]. Given their potential role in regulating toxicological responses, insights into lncRNA function are especially valuable [39]. In line with this, Cao *et al.* applied WGCNA to an integrative single-cell RNA-seq dataset and found that most modules identified in multicell-type scRNA-seq were primarily associated with a single cell type, highlighting strong cell-type specificity [40]. In contrast, Luo *et al.* used a similar approach but identified both cell type–specific and mixed modules, indicating that analyzing multiple cell types together can reveal both distinct and shared networks [41]. These findings collectively suggest that single-cell co-expression analysis can reveal both cell type–specific modules reflecting intercellular changes and mixed modules representing intercellular processes.

To further dissect intercellular co-expression patterns, Panwar *et al.* developed a multicell-type WGNCA (mWGCNA) approach [42]. This method integrates transcriptomes of different cell types by representing each gene with its name plus a cell-type identifier, allowing for the identification of networks containing co-expressed genes from multiple individual cell types. By applying mWGCNA on six distinct cell types from systemic lupus erythematosus (SLE) samples, they identified predominantly mixed modules, underscoring a significant interplay between cell signal-response patterns that are tightly linked, which usually go unnoticed when employing the conventional WGCNA technique across all cell populations concurrently [42].

A similar approach as mWGCNA involves the construction of a multilayer network [43–45], where each layer represents a gene co-expression network for an individual cell type. Here, intralayer edges reflect cell type–specific co-expression, while interlayer edges connect the same gene across different cell types. This approach may aid in the identification of both cell type–specific and more general co-expression patterns.

In summary, direct co-expression analysis of integrated single-cell data from multiple cell types can uncover both cell type–specific patterns and those spanning multiple cell types (Table 1). In toxicogenomics, this can be particularly interesting, as responses to toxicants can vary across cell types. Analyzing both cell type–specific and shared gene expression patterns provides a deeper understanding of how toxicants impact not only cellular responses, but also how different cell types interact, which is crucial for multi-cell-type test systems such as organoids.

#### Uncovering co-expression linked to dynamic cellular phenotypes

Although disease progression can involve distinct cell lineages, pathogenesis can also depend on the conversion of cells from one phenotype to another. For example, conversion of hepatic stellate cells from a resting state to an active myofibroblast phenotype is a key event in progress to liver fibrosis [46, 47]. Applying the methods discussed above to identify networks associated with different cell states (such as healthy and diseased cells) can offer valuable insights into mechanisms of pathogenesis (i.e. phenotype-specific modules) (Fig. 2A_II_). This approach can also be applied to uncover networks that remain consistent across different cell states (i.e. mixed modules), thereby enhancing our understanding of core regulatory processes spanning diverse cellular conditions and cell populations [19].

Several studies illustrate the value of simultaneous co-expression analysis of single-cell or single-nucleus RNA-seq data across multiple phenotypes—including developmental stages [48, 49], disease states [50, 51], or treatment resistance states [52]. These studies have identified phenotypic co-expression networks tightly associated with specific cellular state transitions, underscoring the largely state-specific nature of modules in this approach (Table 1).

However, a serious limitation of available scRNA-seq techniques lie in the isolation procedure itself as the extensive manipulation required can impact mRNA expression levels [53]. In contrast, LCM-RNA-seq avoids this by microscopically excising small histology-defined regions. Co-expression studies on LCM material often recover mixed gene modules spanning several cell states or microenvironments [54, 55]; a reminder that even anatomically focused punches can still contain heterogeneous cell mixtures [56]. High-density spatial transcriptomics extends the idea to thousands of barcoded spots across an intact section [57]. To illustrate, in a murine myocardial-infarction atlas, Yamada *et al.* identified a border-zone module enriched for mechanical stress-response genes that was absent in remote myocardium and linked it, via matched single-nucleus RNA-seq, to stressed cardiomyocytes at the lesion edge [58].

Beyond classical WGCNA, spatially aware frameworks such as hdWGCNA (spot-level WGCNA with spatial mapping) [59], SpaceX (Bayesian spatial Poisson model) [60], and SpaGRN (regulon-based networks) [61] explicitly incorporate neighborhood information, allowing researchers to distinguish modules shared across the tissue from those confined to micro-niches or specific cell types [56].

In conclusion, scRNA and, especially, high-resolution spatial transcriptomics can resolve transcriptional programs at true cell-state level, so co-expression analyses (e.g. WGCNA) tend to yield modules that map cleanly to individual cell states. LCM, while dissociation-free, still excises small but heterogeneous tissue fragments; modules derived from LCM data therefore often mix signals from neighboring cell types. For toxicogenomics, leveraging the higher specificity of scRNA-seq or spatial transcriptomics makes it possible to (i) uncover co-expression networks tightly linked to adverse outcomes after toxicant exposure and (ii) pinpoint the regulatory shifts that accompany a cell’s transition from healthy to stressed or injured states—insights that are diluted when relying on LCM alone.

#### Identifying intracellular gene co-expression networks for a single cell type

Instead of performing a gene co-expression analysis on single-cell data pooled from multiple cell populations, one can also begin by partitioning the data by cell type, or even subtypes among cells of a common lineage, allowing a focused analysis on a single cell type (Fig. 2A_III_). This approach is key to identify intracellular co-expression patterns and can be used to uncover gene regulatory networks specific for each individual cell type or even cell subtype.

Ye *et al.* first applied spectral clustering—a method that groups cells based on similarity defined by eigenvectors of a similarity matrix [62]—to separate a melanoma scRNA-seq dataset into molecularly distinct subpopulations. For each subpopulation, they selected the differentially expressed genes, built a gene co-expression network, and used a dense subgraph learning algorithm to extract “interactive gene groups.” Gene ontology analysis showed that these subgraphs captured biological processes unique to each melanoma subtype [63]. This study highlights that examining cells at a finer resolution can reveal subtype-specific gene networks that might otherwise remain hidden in bulk or unstratified single-cell analyses (Table 1).

Although such analyses may provide interesting insights, they require many samples to support such detailed segmentation. Additionally, applying gene co-expression analyses directly to single-cell data poses several challenges. For one, scRNA-seq only subsamples a fraction of each cell’s transcriptome and often measures only a limited portion of each transcript, potentially causing a biased representation of gene expression [64, 65]. Furthermore, scRNA-seq data are inherently sparse and frequently contain “technical zeros,” making it difficult to distinguish between true absence of expression and low detection efficiency [66–68]. Additionally, many existing co-expression analysis methods were developed for bulk samples and may not adequately account for the heterogeneity, dropout events, and low capture rates typical of single-cell datasets [19].

To address these challenges, several methods—such as hdWGCNA and scGENA—have been developed to increase coverage and reduce noise by grouping highly similar cells into so called “metacells” [59, 69]. This method groups highly similar single cells and averages them into one “pseudo-bulk” profile, boosting unique molecular identifier depth and ironing out dropouts while still retaining cell-state resolution. While this approach can improve data quality, it requires aggregating many samples, which diminishes the downstream sample size available for network construction. This averaging process also reduces the overall variation in the data, which makes it more difficult to detect subtle co-expression changes, potentially limiting the detection of gene networks.

While some studies have reported consistent results between methods originally developed for bulk data (e.g. WGCNA) and newer single-cell adapted frameworks (e.g. hdWGCNA or scGENA), this observed consistency does not per se indicate that these methods work in single-cell context [70]. Instead, it could suggest that both approaches are subject to similar limitations. A benchmarking study by Chen *et al.* found that most methods that assess gene co-expression patterns showed high rates of false-positive network links, failing to reliably predict robust network structures from scRNA-seq data [71]. This trend persisted even when these methods were specifically designed for single-cell approaches—SCENIC (co-expression + transcription factor motif enrichment to infer regulons) [32], SCODE (ordinary-differential-equation modeling of gene expression dynamics) [72], and PIDC (information-theoretic partial-information decomposition to capture direct links) [73]. Moreover, although some studies identify cell type–specific gene networks in single-cell transcriptome data, others suggest that these datasets generally yield weak co-expression signals, flawed downstream analyses, and inaccurate results [71, 74, 75]. Taken together, these findings indeed imply that the consistent modules identified by WGCNA and hdWGCNA in Salem *et al.*’s study may not truly reflect biological processes; instead, they highlight the methodological shortcomings in capturing reliable gene co-expression networks from single-cell data [70].

#### Recommendations for direct single-cell gene co-expression analysis

To determine whether the observed patterns are truly cell-type specific or shared across multiple cell types—reflecting more “core” biological processes—a preservation analysis can be performed. This analysis evaluates whether networks derived from individual cell populations are specific to a given cell type or are preserved (i.e. maintain co-expression properties) across different cell types. This approach highlights whether the fundamental biological processes or pathways represented by the network can also be found in other cell types or states. In principle, networks that form due to technical flaws inherent to a method, dataset, or model, should be specific to that instance and would not be preserved in different datasets using different biological samples. Thus, preservation analysis can help identify reproducible and biologically robust networks and interpretations.

In summary, gene co-expression analyses using single-cell transcriptomics offer valuable insights into both inter- and intracellular co-expression patterns. While single-cell approaches are highly informative, their practical application is often limited by high costs, noise, and data sparsity. In the next section, we explore how bulk RNA-seq data can be leveraged to obtain cell type–specific co-expression patterns, while accounting for sample heterogeneity caused by variability in cell-type composition.

### Indirect approach: deconvolution to address bulk tissue heterogeneity

Disease may begin with initiating events at the level of a single cell type, but progression involves interactions among multiple cell types in a complex tissue environment. Analysis of bulk tissue RNA-seq data represents gene expression from multiple cell types in various proportions and reflect complex interactions among cell types *in situ* across all tissue domains [76]. An approach to address the challenge of extracting cell-specific information from heterogeneous RNA-seq data from bulk tissue samples is to correct or normalize the data for cell-type abundance before applying a gene co-expression analysis. Cell-type abundance in bulk tissue can be estimated *in silico* (Fig. 2B_IIa_) by first leveraging a gene expression signature matrix, obtained from single-cell transcriptomics or by the expression of available cell-type marker genes. These cell-type marker genes may be identified through single-cell analyses (Fig. 2B_Ia/c_) or sourced from publicly available databases such as the Human Cell Atlas (Fig. 2B_Ic_) [77]. Alternatively, the expression levels of the top marker genes for each cell type can also be used directly to estimate cell-type proportions in bulk tissue transcriptomics data (Fig. 2B_IIb_). These obtained cell-type proportions can then be applied in a downstream analysis, e.g. to correlate with established gene co-expression modules from bulk RNA-seq to find modules associated with a certain cell type (Fig. 2B_IIIa_) data or as a confounding factor in the normalization step (Fig. 2B_IIIb_). Numerous studies have shown that deconvolution of bulk tissue gene expression data leveraging markers improves the detection of differentially expressed genes and minimizes the occurrence of false positives and false negatives when compared to results obtained from heterogeneous bulk samples [78]. To minimize the impact of cell-type composition in bulk RNA-seq samples and to identify cell type–specific co-expression patterns, a multistep approach is typical.

#### Step 1: Determine cell-type marker genes

The first step involves identification of cell type–specific marker genes or networks/modules. Individual marker genes can be done by sourcing publicly available resources (Fig. 2B_Ic_). Alternatively, single-cell transcriptomics can be leveraged to discover cell type–specific marker genes, using a signature matrix approach, or cell type–specific networks (Fig. 2B_Ia/b_). For example, McKenzie *et al.* conducted a gene co-expression analysis on single-cell data from both human and mouse brains, and identified co-expression networks enriched with cell type–specific markers [79]. By clustering genes based on their expression profiles, this approach not only confirms known markers, but also uncovers additional candidates through the guilt-by-association principle. Although only Gene7 and Gene8 are published as markers for the “green” cell type (Fig. 2B_Ic_), examining the co-expression module containing these genes also contains Gene10 and Gene23 (Fig. 2B_ib_), suggesting they may also serve as biomarkers for that specific cell type.

#### Step 2: Obtain cell-type proportions

##### Estimate proportions using expression levels of marker genes

The expression levels of the top marker genes can then be used to support the deconvolution of bulk tissue samples (Fig. 2B_IIb_). This approach relies on the principle that, as the proportion of a cell type varies across individuals, the expression of its marker genes will vary accordingly. Consequently, the more specific a marker gene is to a particular cell type, the more precisely its expression levels reflect that cell type’s proportion [80]. As an example, McKenzie *et al.* compared gene expression and immunohistochemistry quantification data and were able to confirm the reliability of the identified marker genes in estimating the cell-type proportions within tissue samples [79]. These findings altogether indicate that unperturbed datasets can be useful to define marker genes that can later be applied for bulk deconvolution methods.

##### Estimate proportions using *in silico* methods

Recent advancements in bioinformatics have paved a way for *in silico* methods to estimate cell-type proportions in bulk tissue data (Fig. 2B_IIa_). Deconvolution algorithms assume that the expression profiles of individual cell types are known or can be approximated using reference datasets or marker gene sets. By leveraging linear regression or machine learning techniques, deconvolution algorithms infer the relative proportions of different cell types within heterogeneous samples. Popular deconvolution algorithms such as CIBERSORT [81], DAISM-DNN^XMBD^ [82], MuSiC2 [83], and MIND [76] have been applied to estimate cell-type proportions and have demonstrated their effectiveness in diverse biological contexts [84–87].

##### Estimate proportions using module eigengene scores

Although the number of studies using single-cell data are increasing, scRNA-seq remains expensive compared to conventional bulk tissue RNA-seq methods and is not always feasible. Kelley *et al.* addressed this problem by pinpointing core transcriptional features that define cellular identities from bulk tissue data and then validating the identified cell-identity signatures with genes sets of cell type–specific marker genes (Fig. 2B_IIc_). This strategy aligns with the observations from Qui *et al.*, who found that marker genes tend to be highly correlated in bulk tissue because their expression varies with the cell type’s proportion [88]. Therefore, when many tissue samples are analyzed in a gene co-expression analysis, these marker genes commonly cluster into modules associated with a specific cell type (Gene1–2 and Gene6–7 in Fig. 1D i). The expression patterns of these high-fidelity genes can then be used to infer variation in the relative abundance of a cell type over heterogeneous samples, which can then be applied in a computational model where gene expression is a function of variation in cellular composition.

The premise of this “top-down” strategy is illustrated by generating synthetic bulk brain samples from different types of scRNA-seq data from the adult human brain for a simulation of the heterogeneity of intact tissue samples [88]. In each of the synthetic datasets, unsupervised gene co-expression analysis was carried out to identify gene co-expression networks, or modules, that were maximally enriched with published markers. The first principal component of these cell-class marker modules—also called the module eigengene score—explains the most variation in a module. This reasoning suggests that a cell-class module eigengene should give an estimation for the relative abundance of that specific cell-class in each sample. Since the exact composition of each synthetic sample was known, they were able to confirm that the actual cellular abundance was indeed nearly identical to that estimated by cell-class module eigengenes.

#### Step 3: Apply cell-type proportions in downstream analysis

The acquired cell-type proportions can be utilized to establish correlations with various factors, such as clinical traits. This enables the exploration of relationships between cellular composition and phenotypic characteristics. In a gene co-expression analysis, a similar method can be applied to characterize cell type–specific gene regulation. In recent studies, cell-type proportions of diverse immune cells in bulk tissue samples were estimated [89–92]. These estimated fractions were then correlated with the established co-regulated gene networks, allowing for a more accurate characterization of the cell type–specific gene regulation of immune cell subpopulations (Fig. 2B_IIIa_). Similarly, Zhong *et al.* generated gene co-expression networks using bulk tissue RNA-seq data of Alzheimer’s patients and applied a multiple regression model incorporating cell-type proportions estimated by CIBERSORT to associate these proportions with module scores [93]. Notably, they found that key Alzheimer’s biomarker (ATN) and non-ATN modules were significantly associated with the expression profiles of various blood cell types. These findings altogether highlight that cell-type proportions can help identify gene co-expression networks that are associated with specific cell types (Table 1).

An alternative approach is to incorporate cell-type proportions into the normalization process. Here, gene counts are regressed on the inferred proportions, and the residuals—now largely free of composition effects—are carried forward to differential or network analyses [94, 95] (Fig. 2B_IIIb_). Several studies have shown that this “composition-corrected” matrix can dramatically reshape single-gene differential expression results. Additionally, Lin *et al.* found that once blood transcripts were corrected for immune-cell fractions, the eigengene profiles of their WGCNA modules no longer differed between patient groups, implying that the apparent module shifts were driven mainly by cell-mix changes (Fig. 1D ii) [94].

Incorporating information about cell-type proportions offers an approach to characterize gene modules exhibiting cell-type gene expression patterns without the need to generate expensive single-cell data. Moreover, applying adjustments to normalize bulk tissue RNA-seq data for cell-type proportions can improve the robustness and interpretability of downstream analyses, as the expression levels are less influenced by the heterogeneity of cell-type proportions. These approaches enable the identification of molecular signatures and regulatory networks that are closely linked to particular cell types, facilitating a deeper understanding of cellular heterogeneity and its functional implications in biological systems.

## Discussion

Gene co-expression profiles derived from bulk tissue RNA-seq data inherently reflect a mixture of different cell types. It is therefore essential to acknowledge and address the influence of cellular heterogeneity when interpreting these results. Herein, we address two main strategies to address this issue. The first involves using scRNA-seq data, where one can either analyze separate cell types or combine multiple cell states and types. The second approach utilized bulk tissue RNA-seq data but corrects for estimated cell-type proportions. We have summarized the strengths and weaknesses of these methods; in the following, we highlight their application to studies on mechanisms of toxicity and pathogenesis caused by exposure to drug and chemical compounds.

Although single-cell gene co-expression analysis allows one to identify cell-type-specific networks and gain a more precise view of each cell’s molecular profile, several limitations may limit its use in mechanistic toxicology studies. First, the process of isolating individual cells can introduce technical artifacts that might impact the observed gene expression profiles. Various studies have identified a significant enrichment of stress response genes in scRNA-seq data, potentially arising from the cellular preparation methods [95, 96]. In the field of toxicogenomics, which primarily aims to evaluate the safety of chemicals by analyzing gene expression profiles, it is noteworthy that the identification of stress responses generally suggests chemical toxicity induced by compound exposure. If these responses stem from the cellular isolation procedures preceding the gene expression profiling, they may be misinterpreted as chemical induced toxicity, increasing the risk of false-positive findings. Although scRNA approaches are expensive, inclusion of transcriptomic data from cells isolated from control tissue can help establish a proper baseline. However, it also complicates the measurement of genuine stress responses because control cells may already exhibit activation due to the isolation protocol. Second, single-cell approaches are limited in feasibility and cost-effectiveness for transcriptome studies that require large sample sizes. In toxicogenomics, it is common to study the effect of different treatments, doses, and time points to characterize gene expression changes linked to toxicological events. Indeed, longitudinal information is an essential part of elucidating time-dependent events in a pathogenic progression. Larger sample sizes and a broader range of conditions are known to enhance co-expression network resolution and allow for a more precise identification of condition-specific modules [18, 97]. However, capturing this complexity with single-cell approaches can be costly. Third, the read depth and gene coverage of scRNA approaches is limited to highly expressed genes, which prohibits a full understanding of the activation of signaling programs that are only detected in whole transcriptome datasets. This might lead to false interpretation of the actual adverse mode-of-action for sufficient accurate safety assessment.

Notably, a gene co-expression analysis on single-cell data generally yields a low number of modules compared to the number of modules generated by bulk tissue. As an illustrative case, Salehi *et al.* conducted an extensive analysis including 33 000 scRNA-seq individual cell samples but found only six co-expression modules [48]. In contrast, conventional gene co-expression studies in toxicogenomics leveraging ~800 bulk tissue whole transcriptome RNA-seq samples typically result in the identification of ~400 modules [15, 16]. Similarly to scRNA-seq, studies applying gene co-expression analysis to LCM data have also yielded a relatively limited number of modules [54, 55]. Therefore, this limitation cannot solely be attributed to the noise in single-cell data. Rather, it suggests that the restricted number of modules may arise from the low variability of single-cell data compared to bulk tissue data and will be determined by the diversity of biological perturbations across the cells and different tissue samples analyzed. Consequently, the model’s ability to capture a diverse range of distinct biological responses might be compromised, resulting in a constrained number of co-expression modules. This assumption is supported by studies where gene co-expression analyses were applied to bulk tissue RNA-seq samples with minimal data variation—in contrast to toxicogenomic studies where increased gene expression is associated with severe injury and has a higher effect size for predicting adverse outcomes [98]. For example, in a study by Tian *et al.*, which incorporated 443 breast cancer samples into a co-expression model, a total of nine modules were found [99]. These findings underscore the importance of a diverse range of conditions within the data to maximize the capture of various biological responses. Large datasets derived from various toxicants with different biological mode-of-actions is ideal to provide such a diversity.

Given the challenges of using scRNA-seq data for gene co-expression analysis in toxicogenomics, a more practical approach might be to use bulk tissue RNA-seq. However, as extensively highlighted in this review, bulk tissue RNA-seq reflects the average expression across all cell types; it can obscure cell type–specific gene co-expression patterns [23–25]. In toxicogenomics analyses, this limitation can have serious implications, as a stress response in a specific cell type can trigger severe adverse effects throughout the entire organ, potentially leading to loss of function. For example, Miao *et al.* identified a significant upregulation of the *Shroom3* transcript in kidney tubule cells of mice, which was linked to fibrosis [100]. This critical finding might be overlooked when using bulk tissue RNA-seq, as the overall expression of *Shroom3* in the kidney is relatively low [101]. To address the problem of cell-type heterogeneity in bulk tissue data, cell-type proportions can be estimated from bulk tissue RNA-seq samples. Various studies have shown that obtaining these cell-type fractions can aid in the detection of cell type–specific co-regulated gene networks either by (1) correcting the data using the cell-type proportions as confounding factors or (2) associating modules with the estimated cell-type proportions, e.g. by applying regression models.

Beyond cell-type composition, temporal dynamics introduces another layer of complexity to both single-cell and bulk toxicogenomics data. Most co-expression studies treat each sample as a static snapshot, even though toxicant responses unfold dynamically over time. Capturing these dynamics requires not only time-aware analytical approaches, but also datasets with sufficient temporal resolution. In single-cell studies, methods such as pseudotime ordering or dynamic community detection can trace evolving gene relationships across exposure trajectories [102–104]. For bulk RNA-seq, temporal co-expression patterns can be uncovered using frameworks that assess changes in gene–gene correlations or by constructing networks across successive time intervals [105–107]. Such strategies benefit greatly from datasets designed with dense time points, as they improve the detection of modules whose connectivity patterns change during the response. Embedding temporal resolution into both study design and analysis will be essential to distinguish short-term adaptive responses from molecular changes that persist and potentially drive progression toward an adverse outcome.

Cell type–specific co-expression analysis plays a crucial role in toxicogenomics, where understanding how different cell types respond to toxicants can provide insights into the mechanisms of toxicity and help to predict adverse outcomes. In simple cell models, such as RPTEC-TERT1—an immortalized kidney epithelial cell line—or primary human hepatocytes (PHH), assessing cell type–specific co-expression might not be necessary, as the system consists of a homogeneous cell population with uniform responses [15]. However, in more complex test systems like organoids derived from induced pluripotent stem cells, the importance of cell type–specific co-expression becomes evident. Such organoids contain a mix of cell types with different functions and varying proportions, and some cell types might be present in very low numbers [108]. Toxicants can affect each cell type differently, and without assessing cell type–specific co-expression patterns, key cellular responses underlying organ-level toxicity could be missed. Therefore, as the complexity of the test system increases, so does the need for refined approaches to accurately capture the interactions between different cell types, particularly those in low abundance, and their role in toxicity responses. The choice whether to perform a gene co-expression analysis on bulk tissue (either corrected for cell-type proportions or not) or single-cell data depends on the specific research goal. Both approaches have their advantages and disadvantages, and the choice ultimately depends on the experimental design and available resources.

**Key Points**

- Bulk RNA-sequencing signals are inherently influenced by varying cell type compositions, making it essential to account for this heterogeneity in co-expression analyses.
- Two overarching solutions exist: (1) perform co-expression analysis at the single-cell level or (2) estimate and correct for cell-type proportions in bulk data, or use these estimates to define cell type–specific modules.
- Single-cell approaches can provide more precise cell type–specific insights, but are less scalable for large toxicogenomics studies.
- The choice of method depends on the study’s goals, balancing feasibility and resolution.

## Supplementary Material

SupplementalFigure1_bbaf421
