# Supplementary figures and images for: Cell type heterogeneity in gene co-expression networks: implications for toxicological research
Source: Brief Bioinform. 2025 Aug 26;26(4):bbaf421. doi: 10.1093/bib/bbaf421 (PMC12378898; doi:10.1093/bib/bbaf421)

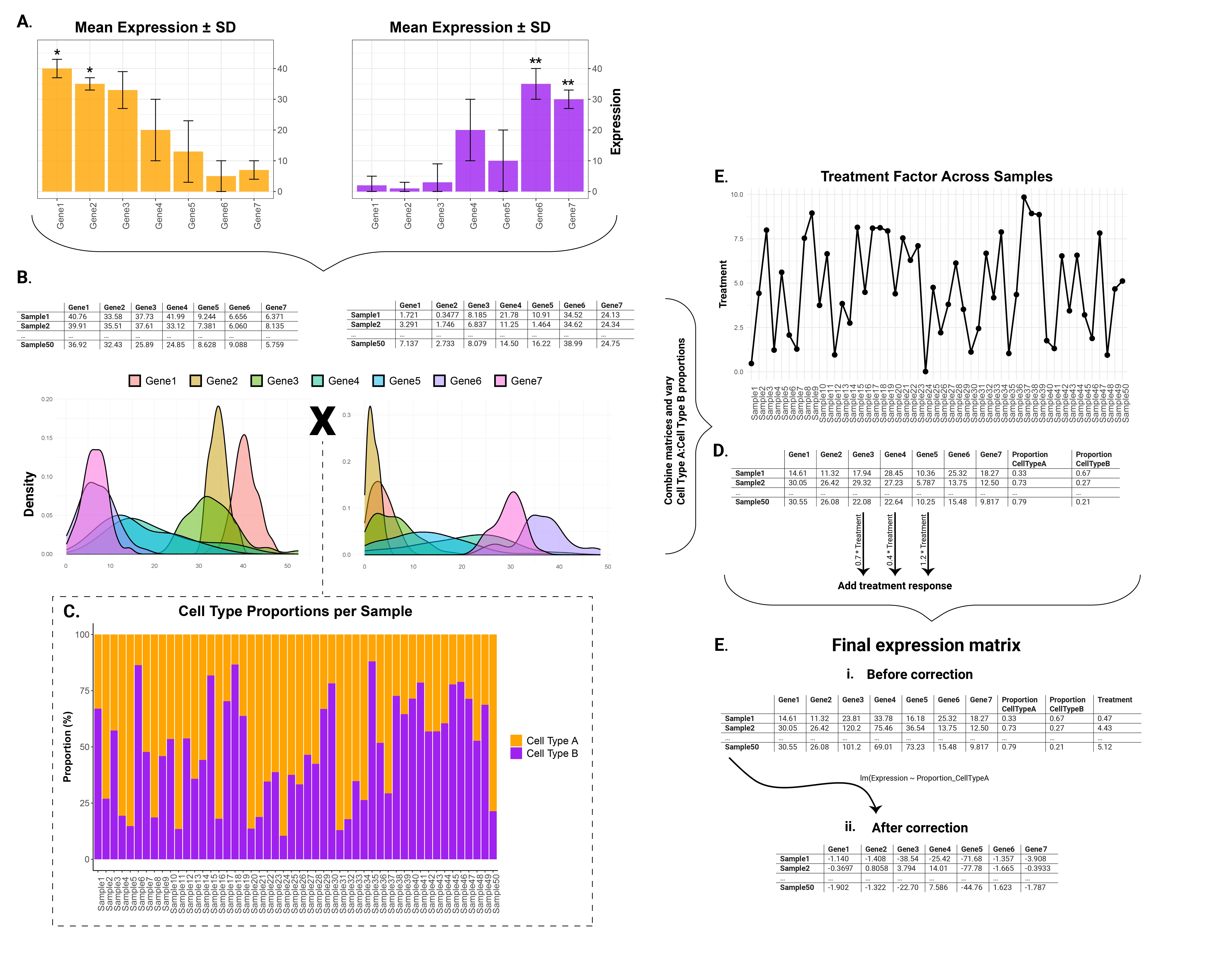

Supplement: SupplementalFigure1_bbaf421 [file supplementalfigure1_bbaf421.jpeg]
